# Human umbilical cord mesenchymal stem cells regulate glutathione metabolism depending on the ERK–Nrf2–HO-1 signal pathway to repair phosphoramide mustard-induced ovarian cancer cells

**DOI:** 10.1515/biol-2022-0997

**Published:** 2024-11-16

**Authors:** Lu Sun, Xiaodong Fan, Qian Chen, Guoyan Liu

**Affiliations:** Department of Gynecology, Tianjin Medical University General Hospital, Tianjin, 300052, China; Department of Gynecology, Tianjin Central Hospital of Obstetrics and Gynecology, Tianjin, 300100, China; Department of Ultrasonography, Tianjin Central Hospital of Obstetrics and Gynecology, Tianjin, 300100, China; Department of Gynecologic Oncology, Tianjin Medical University Cancer Institute and Hospital, Huan hu xi Road, Hexi District, Tianjin, 300060, China

**Keywords:** premature ovarian insufficiency, phosphoramide mustard, human umbilical cord mesenchymal stem cells, oxidative stress, glutathione, reactive oxygen species, ovarian cancer cells

## Abstract

The aim of this study was to study the effects of human umbilical cord mesenchymal stem cells (HUC-MSCs) on glutathione (GSH) metabolism in human ovarian cancer cells induced by phosphoramide mustard (PM). The experiment was divided into five groups, namely, the blank group (ovarian cancer cells), the control group (ovarian cancer cells + HUC-MSCs), the model group (ovarian cancer cells + PM), the treatment group (ovarian cancer cells + PM + HUC-MSCs), and the inhibitor group (ovarian cancer cells + PM + HUC-MSCs + extracellular signal-regulated protein kinase inhibitor PD98059). The apoptosis rate of ovarian cancer cells was detected by flow cytometry. Intracellular levels of oxidized glutathione (GSSG), GSH, γ-glutamyl cysteine synthetase (γ-GCS), and intracellular reactive oxygen species (ROS) were detected by enzyme-linked immunosorbent assay. Protein imprinting and real-time fluorescence quantitative PCR were used to detect extracellular regulated protein kinase (ERK), p-ERK heme oxygenase-1 (HO-1), and nuclear factor E2-related factor 2 (Nrf2) protein levels. First, the apoptosis rate in the model group was increased compared with that of the blank group. The levels of γ-GCS, p-ERK, HO-1, and Nrf-2 decreased, while the levels of malondialdehyde, GSSG, and ROS increased. Second, compared with the model group, the apoptosis rate in the treatment group decreased. GSH, γ-GCS, p-ERK, HO-1, and Nrf2 levels increased. Malondialdehyde, GSSG, and ROS levels decreased. Third, after the administration of ERK inhibitor, the apoptosis rate of cells increased. GSH, p-ERK, and HO-1 levels decreased. GSSG and ROS levels increased (*P* < 0.05), and γ-GCS level had a downward trend compared with the treatment group. To conclude, HUC-MSCs may regulate the ERK–Nrf2–HO-1 pathway to increase γ-GCS expression and GSH production, reduce ROS level and apoptosis of ovarian cancer cells, and improve antioxidant capacity.

## 1 Background

Chemotherapy is one of the important treatment methods for cancer, but the damage to ovarian reserve function caused by chemotherapy in young female patients is receiving increasing attention. At present, there is no effective treatment method for ovarian reserve function damage caused by chemotherapy, which is an urgent clinical problem to be solved. Ovarian reserve dysfunction, also known as premature ovarian insufficiency (POI), develops into premature ovarian failure (POF) in its final stage. POI refers to the amenorrhea that occurs after puberty and before the age of 40. It is accompanied by hot flashes, night sweats, decreased sexual desire, and infertility. One in 250 women will suffer POI before the age of 35, and one in 100 women will become sick before the age of 40 [1,2], which can cause disorders in the endocrine system and increase the risks of osteoporosis and cardiovascular diseases in women.

At present, the main methods for treating POI include (1) hormone replacement therapy, (2) egg donation/recovery and embryo transfer in the hormone replacement therapy cycle, and (3) frozen ovarian transplantation. The first two methods need hormone support, which increases the risks of endometrial cancer, ovarian cancer, and breast cancer. Human umbilical cord mesenchymal stem cells (HUC-MSCs), as a class of immunocompromised cells, do not cause immune rejection in allogeneic or even xenogeneic transplantation and are widely used in research.

Chemotherapy induced POI by many mechanisms, such as (1) hormone receptor signaling regulation and reproductive hormone metabolism regulation; (2) epigenetic alterations; (3) mitochondrial dysfunction; (4) oxidative stress (OS) disorder; and (5) immune function disorder [3]. One of the important mechanisms is OS changing in ovarian cells [3]. The enhancement of OS can induce the apoptosis of granulosa cells, which leads to follicular atresia and damages the quality of oocytes. A lot of papers use cyclophosphamide (CTX) to build cells or animal chemotherapy-induced models to study the reactive oxygen species (ROS) change [4,5,6]. Therefore, we thought that it was meaningful to study OS change in chemotherapy-induced POI.

At present, HUC-MSCs can possibly repair POI as follows: increase the plasma sex hormone level and induce to differentiate ovarian granulosa-like cells [7]; secrete cell growth factors, such as hepatocyte growth factor, vascular endothelial growth factor, and insulin-like growth factor 1 to promote oocyte regeneration [8,9,10]; secrete exosomes [11], improving granulosa cell function through microRNAs (miR), such as miR-99a, miR-100, miR-132, and hsa-miR-130bmRNAs [12,13].

As an important antioxidant, glutathione (GSH) plays a critical role in protecting cells from oxidative damage, the toxicity of xenobiotic electrophiles, and maintaining redox homeostasis [14]. Besides, GSH is widely used for liver protection during chemotherapy. Recombinant glutamate cysteine ligase, catalytic (GCLC) and glutamate cysteine ligase, modifier subunit (GCLM) are the key subunits of γ-glutamyl cysteine synthetase (γ-GCS) which also the downstream proteins of heme oxygenase-1 (HO-1). HO-1 was the downstream protein of nuclear factor E2-related factor 2 (Nrf2) [15]. Extracellular regulated protein kinase (ERK) 1/2 is considered the top biological factor that participates in oxidative and ER stress in the Post-Intracerebral Hemorrhage Stroke Rat Brain model [16]. In some research, the expression of ERK had a positive relationship [17].

We wanted to know whether HUC-MSCs can regulate GSH metabolism depending on the ERK–Nrf2–HO-1 signal pathway to repair the chemotherapy-induced POI model. In 2000, Zhang studied the biological characteristics of the Cov434 cell line. The conclusion was that the Cov434 cell line had similar characteristics to granulosa cells and may be useful for experimental studies on follicular development [18]. Besides, the Cov434 cell line was used in many studies related to granulosa cells, and the CTX-induced Cov434 cell line is considered a chemotherapy-induced POI *in vitro* model [19,20]. Therefore, we use the Cov434 cell line to establish the ovarian cancer model of POI to study the effects of HUC-MSCs on OS level and GSH metabolism of GC line and further to provide a theoretical basis for HUC-MSCs to treat POI.

## 2 Materials and methods

### 2.1 Isolation and culture of HUC-MSCs

Umbilical cords were collected from normal-term primipara fetuses. This work received approval from the Institutional Ethics Committee. The collected umbilical cords were fully washed with phosphate-buffered solution (PBS). Both ends of the umbilical cord, measuring 0.5 cm in length, were removed. Subsequently, the umbilical cord was cut into 2 cm long segments. The umbilical cord was cut longitudinally along the lumen of the umbilical vein, the inner membrane of the umbilical vein was peeled off, and the tissue was cut to a block smaller than 1 mm × 1 mm × 1 mm. The umbilical cord was cultured with a medium that contained 10% fetal bovine serum and 1% penicillin–streptomycin in a carbon dioxide incubator at 37°C, 5% CO_2_, and 95% humidity. After 7 days, the climbing HUC-MSC cells were harvested and separated. For standby, three to five generations were maintained. The identification of cell surface markers CD34, CD44, CD45, CD73, CD90, and CD105 by flow cytometry conforms to the standards of mesenchymal stem cells (MSCs).


**Informed consent:** Informed consent has been obtained from all individuals included in this study.
**Ethical approval:** The research related to human use has been complied with all the relevant national regulations, institutional policies and in accordance with the tenets of the Helsinki Declaration, and has been approved by the Institution Review Board, Tianjin Central Hospital of Obstetrics and Gynecology (No. 2020KY058).

### 2.2 Multiple differentiation potential of MSCs

Add 1 × 10^5^ cells and 1.5 mL cell culture medium to each empty space in a six-well cell culture plate, mix well, and place in a CO_2_ incubator for cultivation. After 24 h of cultivation, the cells were observed to fully adhere to the wall and achieve a fusion rate of over 50% under the microscope. Two wells were replaced with adipocyte induction mediums and two wells were replaced with osteogenic induction medium. Half of the corresponding medium was replaced every other day until significant changes were observed in the induction wells (many oil droplets appeared in the adipose induction wells and many extracellular secretions appeared in the osteogenic induction wells). Oil red O staining solution and Alizarin red staining solution were used to stain adipose induction cells and osteogenic induction cells, respectively, and uninduced well cells were used as controls.

### 2.3 Establish ovarian cancer cell line injured by CTX

We selected an ovarian cancer cell line (FengHui ShengWu, China) as the cell culture for the following test. Previous literature shows that CTX and its metabolite phosphoramide mustard (PM) (Yuanye Shengwu, China) can cause cell damage. We tested the damage effects of CTX and PM on the ovarian cancer cell line (FengHui ShengWu) simultaneously. The experiment was divided into three groups: the blank group (only M5A complete medium, without cells), the negative control group (ovarian cancer cells without drugs), and the experimental group (ovarian cancer cells with different concentrations of drugs). Cells were selected and added to 96-well plates when they reached the logarithmic growth phase, with 100 µL cell suspension and 1,000 cells per well. After 24 h of planking culture, CTX was incubated at concentrations of 0, 5, 10, 20, 50, 100, and 200 µM. The PM was added to the corresponding 96 well plates at concentrations of 2, 5, 10, 20, 50, 100, and 200 µM, and the drug intervention lasted for 7 days.

Because of the excellent performance of PM, we used PM as the cell damage inducer. The cells were divided into five groups: the blank group (only ovarian cancer cells), the model group (ovarian cancer cells + PM), the treatment group (ovarian cancer cells + PM + HUC-MSCs), the inhibitor group (ovarian cancer cells + PM + HUC-MSCs + PD98059), and the control group (ovarian cancer cells + HUC-MSCs).

### 2.4 Cell viability by CKK8 assay

Ovarian cancer cells were plated in a 24-well plate with 1,000 cells per well. MSC cells were plated in 24 transwell chambers. Equal amounts of MSC cells were added to the transwell chambers for injury treatment. The cell culture plate was collected for 7 days. A total of 10 µL cell counting kit-8 (CCK8) solution was added into each hole and incubated in the incubator for 4 h. The absorbance of each hole was measured and calculated at 450 nm using a microplate reader.

### 2.5 Apoptosis assay by flow cytometry

1. Select granulosa cells and MSC cells with logarithmic growth phase, digest them with 1 mL trypsin cell digestion solution, observe under the microscope, and when the cell gap becomes larger and the cells shrink and become round, immediately add 2 mL of complete culture medium to terminate digestion. Gently disperse the cell suspension, transfer it to 10 mL centrifuge tubes using a pipette, centrifuge at 1,000 rpm for 5 min, and discard the supernatant. Wash the cells three times with 3 mL of PBS.
2. Cell count: If the cell density is high, the cells can be diluted 10 times first, and then 10 µL of cell suspension can be added to the cell culture plate for counting. A total of 2,500 cells are added per well to calculate the total volume of cell suspension required for laying the plate.
3. Cell laying: cells are laid with one 24 well plate, and MSC cells are laid into the transwell chamber. A total of 24 transwell chambers are laid, with 2,500 cells all laid.
4. Cell dosing: After 24 h of cell treatment, different groups of dosing treatments were performed, with three parallel wells set in each group, divided into five different treatment groups.

Avarian cancer cell apoptosis was assessed using Annexin V–fluorescein isothiocyanate (FITC) Apoptosis Detection Kit (Beyotime, China). Ovarian cancer cells were collected and washed with PBS. A total of 195 µL Annexin V–FITC binding solution was added to each group. A total of 5 µL Annexin V–FITC and 5 µL propidium iodide were mixed and then detected by flow cytometry detection.

### 2.6 Toxicity of PD98059 on ovarian cancer cells

We applied an ERK inhibitor (PD98059) to inhibit ERK activation and determine the role of the ERK signaling pathway in MSC repair. Before the inhibition test, we need to test the safe dose of PD98059 (MCE, America) on ovarian cancer cells to avoid its lethal effect on ovarian cancer cells. Hence, we tested the inhibition of PD98059 on the growth of cells. The experimental grouping was synchronous with step “Cell model establishment.” The final diluted concentration of PD98059 was 2, 5, 10, 20, 50, 100, and 200 µM. The drug intervention time was 4 days and 7 days. Cell viability was detected by the CCK8 test.

### 2.7 Oxidized glutathione (GSSG), GSH, γ-GCS, ROS, and malondialdehyde (MDA) determination

Experimental reagents were prepared according to the instructions of the kit. A total of 10 mM GSSG stocked solution was diluted to 10, 5, 2, 1, and 0.5 µM GSSG solutions to prepare a standard curve, and the GSSG content in the sample was determined. Cells with PBS were washed. The cells were centrifuged and collected, frozen and thawed twice rapidly, and centrifuged at 10,000 × *g* for 10 min. The supernatant was taken for total GSH determination. Some of the above-prepared samples were taken with total GSH content for measurement. A total of 20 µL diluted GSH scavenging auxiliary solution was added to each 100 µL sample, followed by immediate vertexing and mixing. Then, GSH scavenging reagent working solution at a ratio of 4 µL GSH scavenging reagent working solution per 100 µL sample was added for subsequent determination. Immediately use an enzyme-linked immunosorbent assay reader to measure A412, record as a reading value for 0 min, record every 2 min, and record continuously for at least 10 min. A total of 50 microliters of 0.5 mg/mL nicotinamide adenine dinucleotide phosphate (NADPH) solution was added and mixed well. A405 was measured with a microplate reader once every 5 min or in real time for a total of 25 min. Five data points were also measured. The total GSH and GSSG contents in the sample were calculated.

The cells with PBS were washed, and the cells were centrifuged and collected, frozen and thawed twice rapidly, and centrifuged at 10,000 × *g* for 10 min. DCFH-DA with serum-free culture medium was diluted in a ratio of 1:1,000 to a final concentration of 10 µmol/L. Cells were suspended in diluted DCFH-DA and incubated for 20 min. Cells were washed three times with PBS to remove DCFH-DA for ROS detection. ROS was detected by using a fluorescent probe DCFH-DA by flow cytometry.

ELISA was used to detect levels of GSH, GSSG, γ-GCS, and MDA according to the steps provided in the kit (Beyotime, China).

### 2.8 Real-time fluorescence quantitative PCR analysis

Terminate the culture of five groups. Then extract total RNA from cells of each treatment group. Remove the cell culture plate from the cell culture incubator and immediately process it. Remove the cell culture medium and wash the cells once with PBS. Precooling centrifuge for a marriage proposal at 4°C. Transfer cell lysates from different groups to pre-labeled corresponding 1.5 mL RNA-free EP tubes and let them stand on ice for 5 min. Add 100 μL of chloroform to each tube, mix thoroughly, and let it stand on ice for 10 min to completely dissociate the nuclear protein complex. Centrifuge at 12,000 rpm for 15 min at 4°C. During this period, take a new EP tube, add 500 μL of isopropanol, and precool on ice. After centrifugation, transfer the upper aqueous phase to the new EP tube. Let it stand on the ice and precipitate with ethanol in a −20°C refrigerator for 10–30 min. Centrifuge at 4°C, 12,000 rpm, for 10 min. Remove the supernatant. Wash the RNA precipitate once with 1 mL of 75% ethanol. Centrifuge at 12,000 rpm for 10 min.

Remove the supernatant and air dry RNA precipitation for 5–10 min. Dissolve RNA in 20 μL of deionized water treated with DEPC. Perform spectrophotometric analysis to determine sample concentration and purity. Reversed transcribe the total RNA extracted from each group of cells into cDNA according to the instructions of the reverse transcription kit. Use real-time fluorescence quantitative PCR to detect γ-GCS mRNA’s, ERK’s, HO-1’s and Nrf-2’s expression.

### 2.9 Western blotting analysis

Cells were harvested and digested by pancreatic enzymes. Protein samples were extracted after 30 min of incubating the mixture on ice. The protein samples were measured by a BCA protein quantitative kit for concentration and quantification, followed by gel preparation, electrophoresis, membrane transfer, and sealing. The membranes were incubated with primary antibody against Nrf2, HO-1 (Sigma, America), ERK, p-ERK (CST, America), and β-actin (Affinity, America) overnight. On the next day, HRP-labeled rabbit secondary antibody was added to the membranes and incubated for 1 h. Finally, it was developed and analyzed by a Western blot developer.

### 2.10 Statistical analysis

Data were analyzed by SPSS 25.0 statistical software. The measurement data were expressed as mean ± standard deviation (*x* ± *s*). Independent-samples *t*-test was used to analysis of differences between two groups with a statistical significance set at *p* < 0.05.

## 3 Results

### 3.1 Successful isolation, culture, and identification of HUC-MSCs

**Figure 1:**
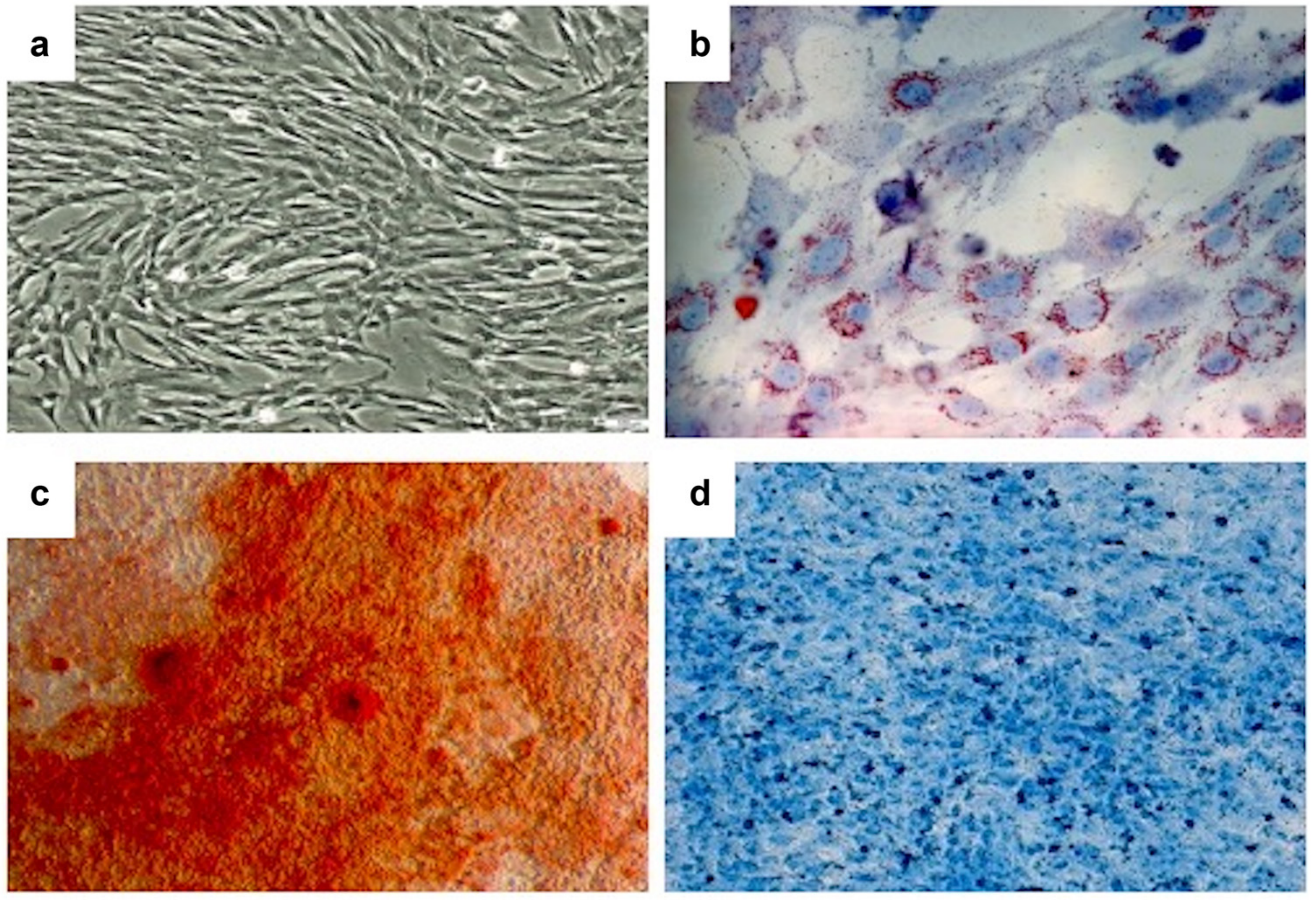
Morphology and multiple differentiations of HUC-MSCs isolated *in vitro*. (a) Morphology of HUC-MSCs; (b) adipogenic differentiation of HUC-MSCs; (c) osteogenic differentiation of HUC-MSCs; and (d) cartilage formation differentiation of HUC-MSCs.

**Figure 2:**
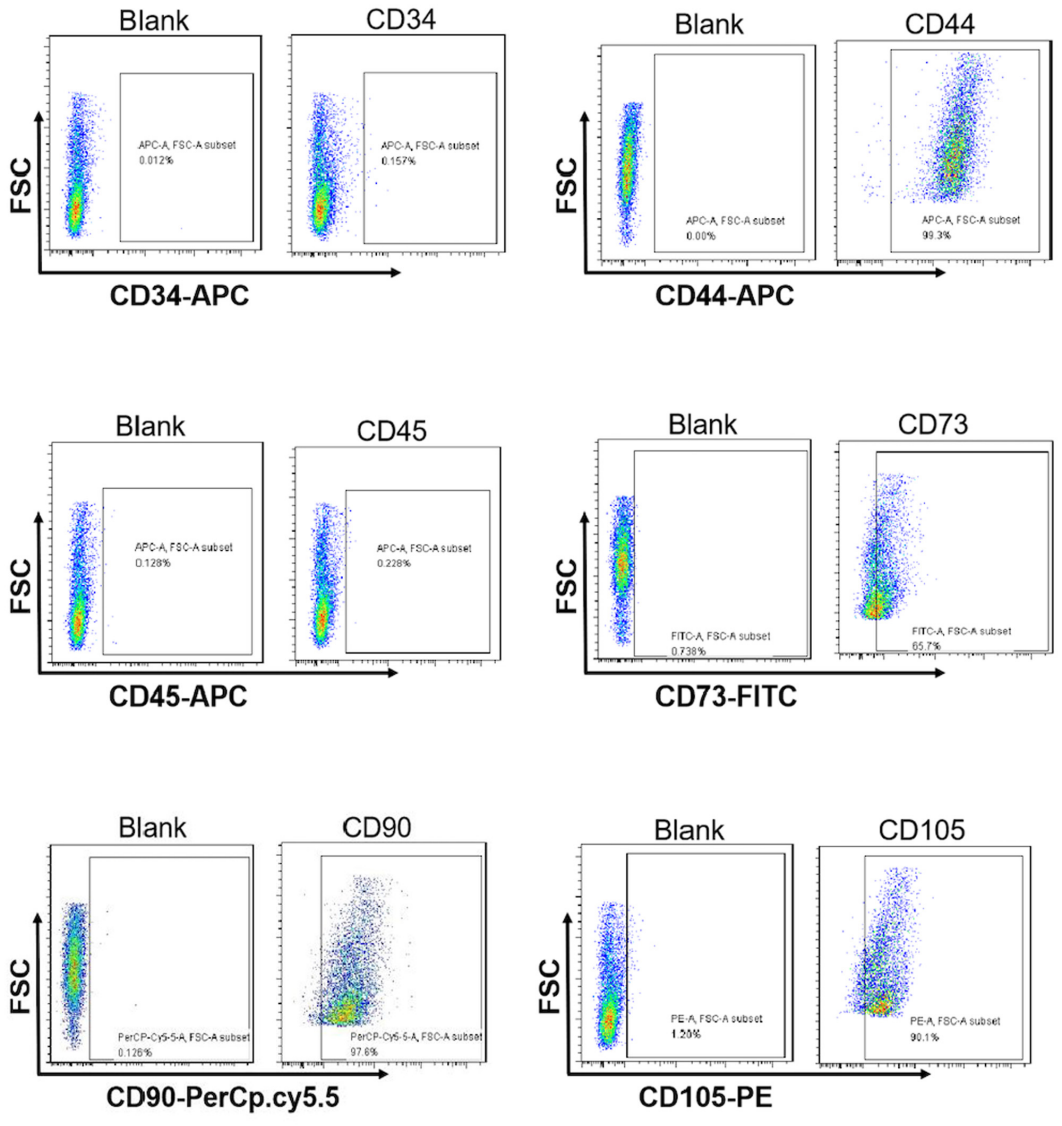
Analysis of surface markers on HUC-MSCs by flow cytometry.

HUC-MSCs could grow stably after the primary passage, and the cell morphology, which was spindle-shaped or fibroblast-like, was uniform. HUC-MSCs have been cultivated for different generations. P1 means the first generation, and P2 means the second generation. (Figure 1a). HUC-MSCs induced into adipocytes, osteoblasts, and chondrocytes (Figure 1b–d). The flow cytometry results showed that the HUC-MSCs isolated in this experiment were CD34– CD45– CD105+ CD90+ CD44+ CD73+ cells and the markers on the surface of HUC-MSCs were correct, which could be used in future experiments (Figure 2).

### 3.2 Establishment of cell injury model

**Figure 3:**
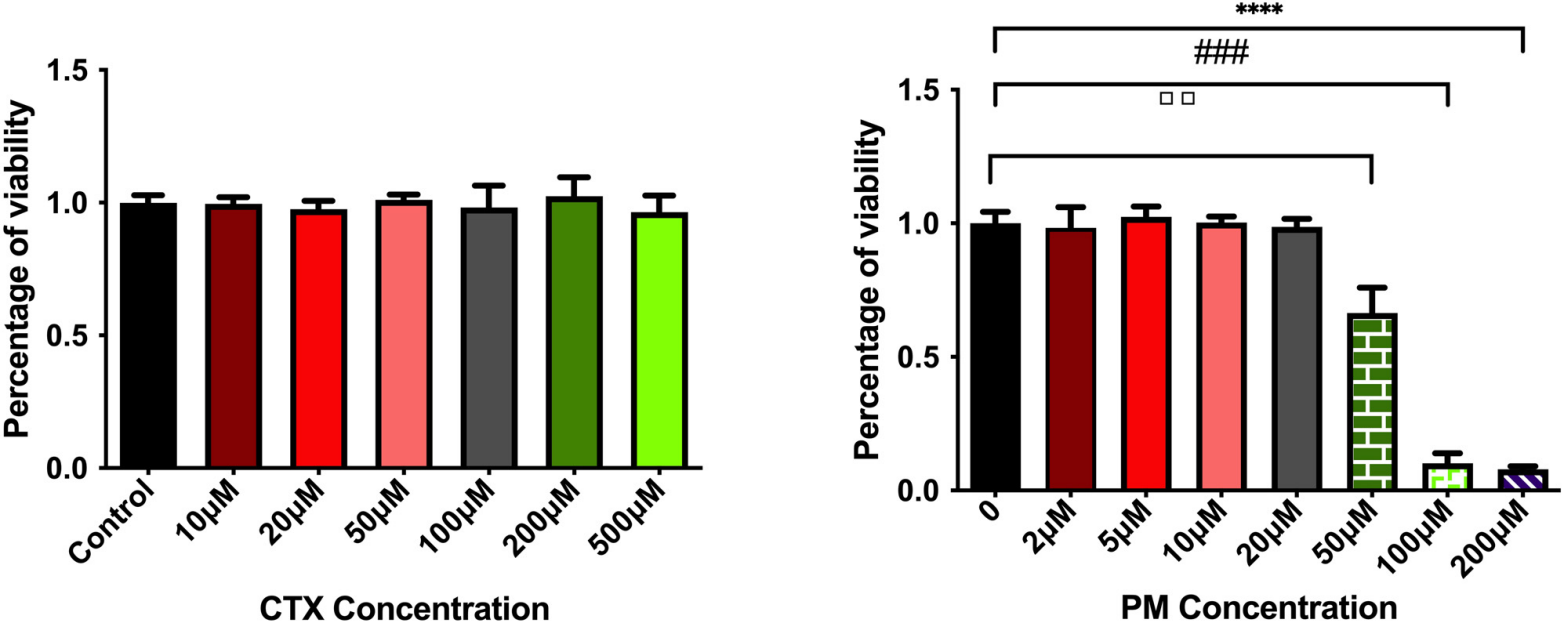
Effects of different concentrations of CTX and PM on the viability of granulosa cells. ***P < 0.0001 vs 0 mM, ^###^ *P* < 0.0001 vs 0 mM, ▫▫*P* < 0.001 vs 0 mM.

The results showed that the activity of ovarian cancer cells had no statistical difference in each concentration of the CTX-treated group compared with the negative control group. However, PM could inhibit the proliferation of cells in a concentration-dependent manner and completely inhibit the growth of ovarian cancer cells at 50 and 200 µM, and the difference was statistically significant (*P* < 0.05) (Figure 3). Hence, CTX was an active potential drug that must be oxidized and metabolized further into its active metabolite *in vivo* to play its role. Therefore, we use the PM active metabolite of CTX for experimental research in later experiments.

### 3.3 HUC-MSCs could inhibit PM-induced apoptosis of ovarian cancer cells

**Figure 4:**
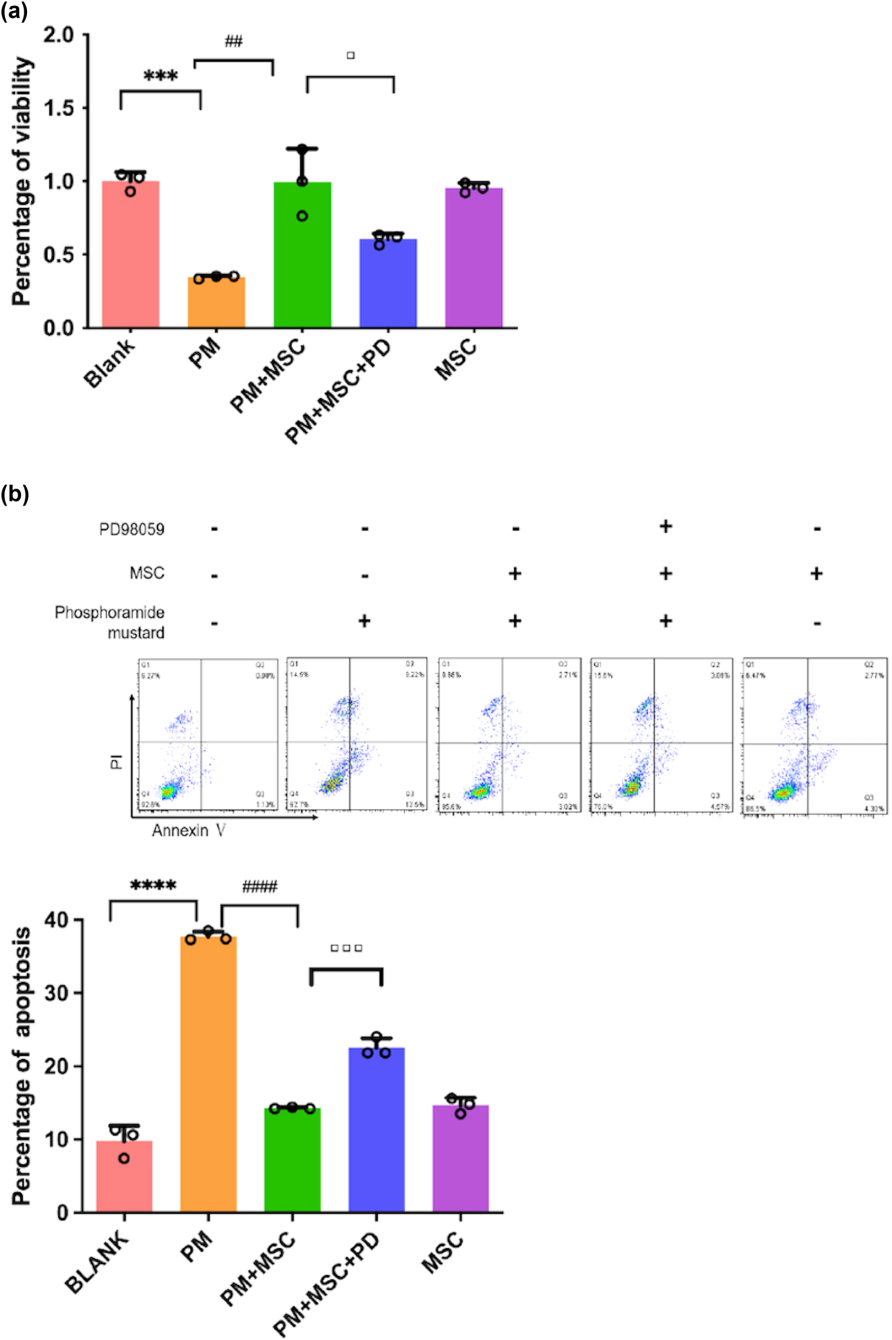
The viability of ovarian cancer cells in different groups. (a) The viability of ovarian cancer cells by CCK8 test, ***P < 0.001 vs blank group, ^##^ *P* < 0.01 vs PM group, ▫*P* < 0.05 vs PM + MSC group. (b) Apoptosis of ovarian cancer cells in different treatment groups by flow cytometry. ****P < 0.0001 vs blank group, ^####^ *P* < 0.0001 vs PM group, ▫▫▫*P* < 0.001 vs PM + MSC group.

CCK8 test showed that PM could significantly inhibit the survival rate of ovarian cancer cells and the difference was significant (*P* < 0.05) compared with the blank group (Figure 4a). Compared with the model group, the damaging effect of ovarian cancer cells was significantly repaired, and the cell survival rate was significantly increased after co-culturing with HUC-MSCs (*P* < 0.05) in the Boyden chamber transwell. Flow cytometry detection of apoptosis also confirmed that the apoptosis rate in the treatment group was lower than that in the model group, with a statistically significant difference (*P* < 0.01) (Figure 4b).

### 3.4 HUC-MSCs reduced the ROS level in Ovarian cancer cells induced by PM

**Figure 5:**
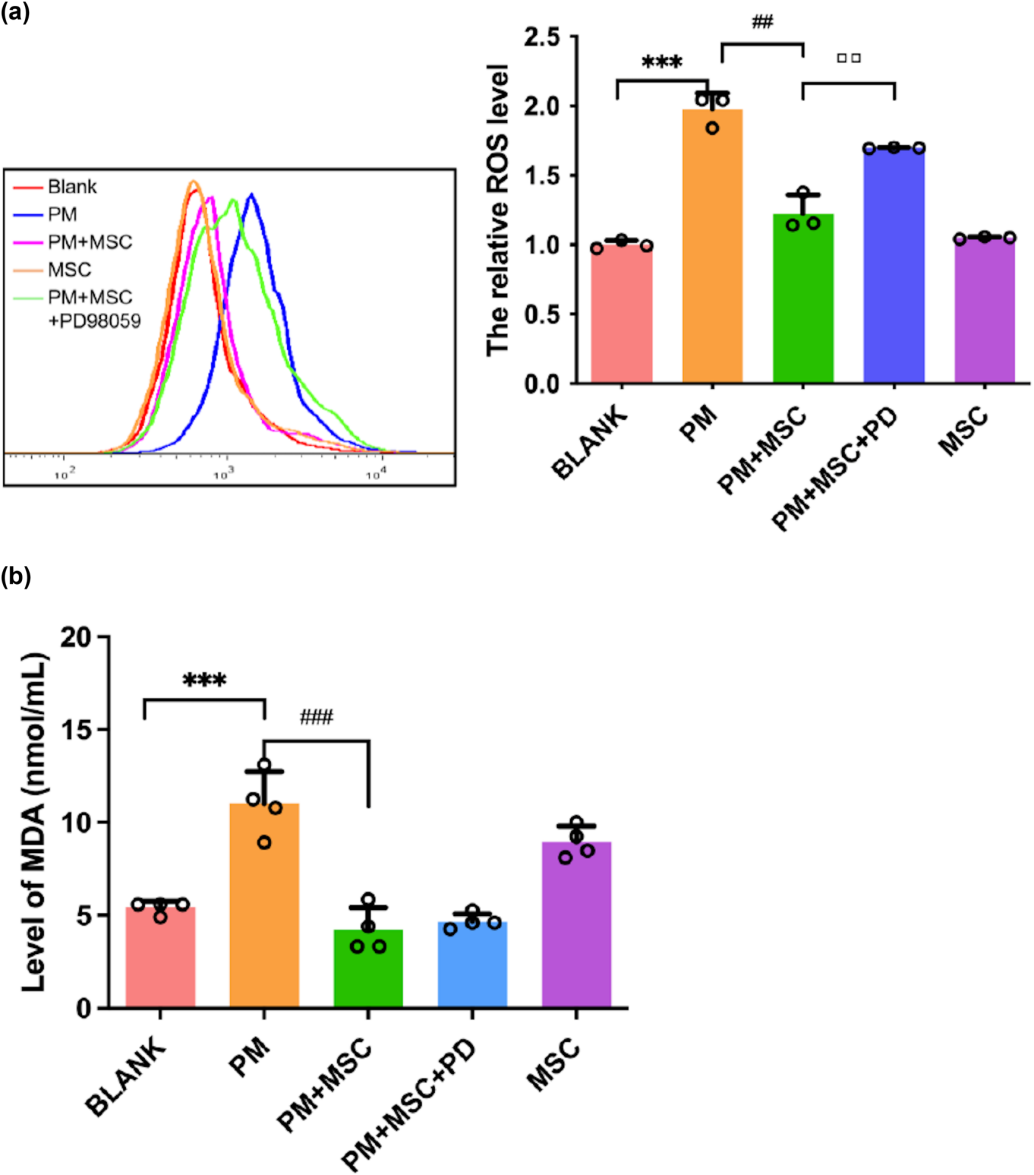
The levels of ROS and MDA in granulosa cells of different treatment groups. (a) The level of ROS in granulosa cells of different treatment groups by flow cytometry. ***P < 0.001 vs blank group, ^##^ *P* < 0.01 vs PM group, ▫▫*P* < 0. 01 vs PM + MSC group. (b) The level of MDA in Cov434 cells in different treatment groups. ***P < 0.001 vs blank group, ^###^ *P* < 0.001 vs PM group.

Compared with the blank group, the ROS and MDA levels in ovarian cancer cells in the model group increased significantly (*P* < 0.01). After HUC-MSC treatment, the ROS level was decreased compared with the model group (*P* < 0.05), and the level of MDA tended to decrease. This finding indicated that HUC-MSCs had significant repair and clearance effects on abnormal ROS generated by PM-induced damage to ovarian cancer cells (Figure 5a and b).

### 3.5 HUC-MSCs increased GSH levels and decreased GSSG levels in ovarian cells through the expression increase of γ-GCS

**Figure 6:**
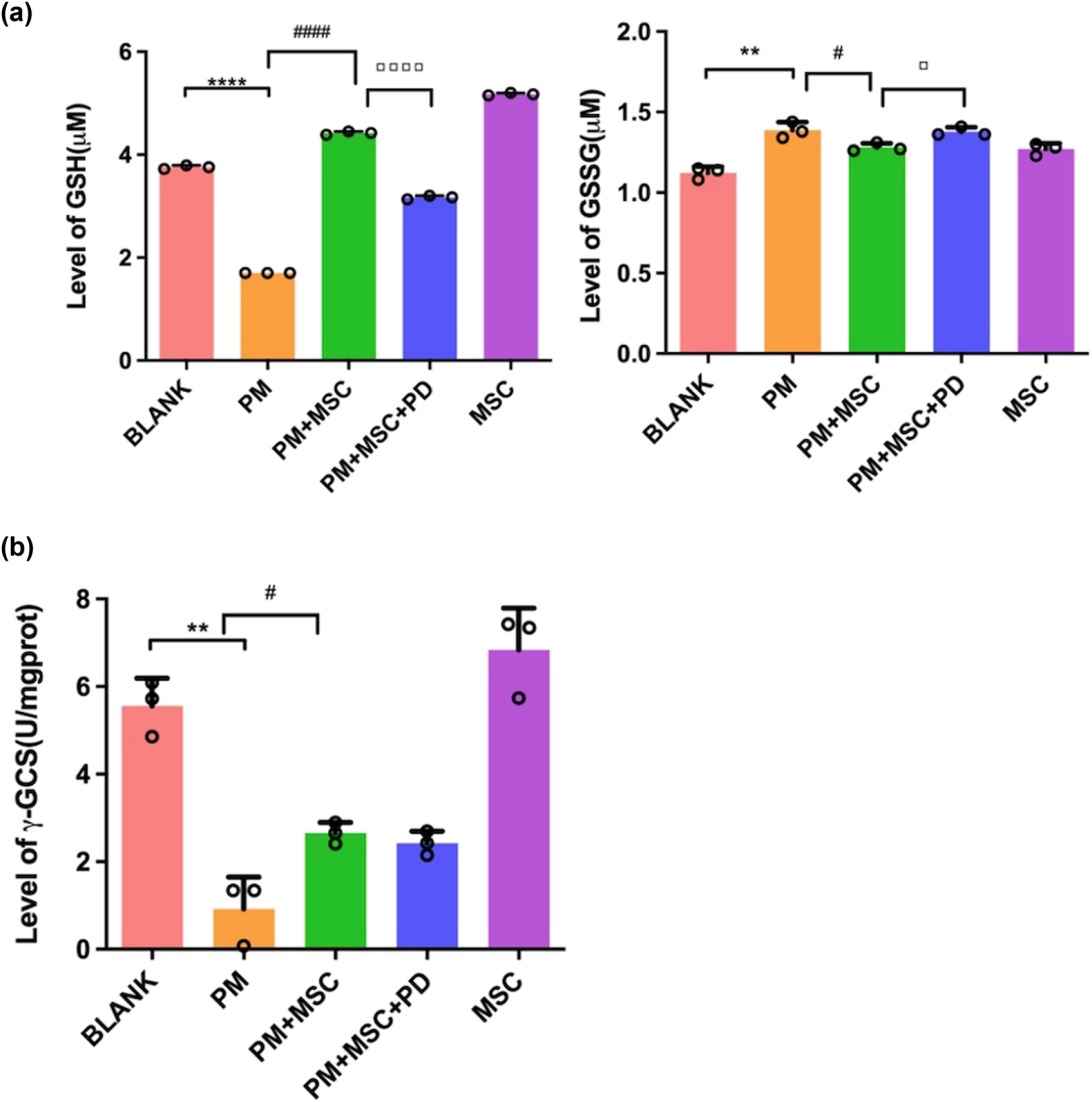
HUC-MSCs increased GSH levels and decreased GSSG levels in granulosa cells by increasing the expression of γ-GCS. (a) Levels of GSH and GSSG in ovarian cancer cells in different treatment groups, ****P < 0.0001, **P < 0.01 vs blank group, ^####^ *P* < 0.0001, ^#^ *P* < 0.05 vs PM group, ▫▫▫▫*P* < 0.0001, ▫*P* < 0.05 vs PM + MSC group. (b) Levels of γ-GCS in ovarian cancer cells in different treatment groups. **P < 0.01, vs blank group, ^#^ *P* < 0.05 vs PM group.

Compared with the blank group, the expression level of GSH in ovarian cancer cells in the model group was significantly lower (*P* < 0.01) while the level of GSSG was significantly higher (*P* < 0.05). After co-culturing with HUC-MSCs cells, the expression levels of GSH increased significantly (*P* < 0.01), whereas the level of GSSG decreased compared with the model group (*P* < 0.05) (Figure 6a).

γ-GCS is the rate-limiting enzyme in the step of GSH synthesis. Compared with the blank group, the activity level of γ-GCS in the model group was decreased (*P* < 0.05). After co-culturing with HUC-MSCs, the level of γ-GCS in cells increased significantly compared with the model group (*P* < 0.05) (Figure 6b).

### 3.6 The toxicity of PD98059 on ovarian cancer cells detected by CCK8

**Figure 7:**
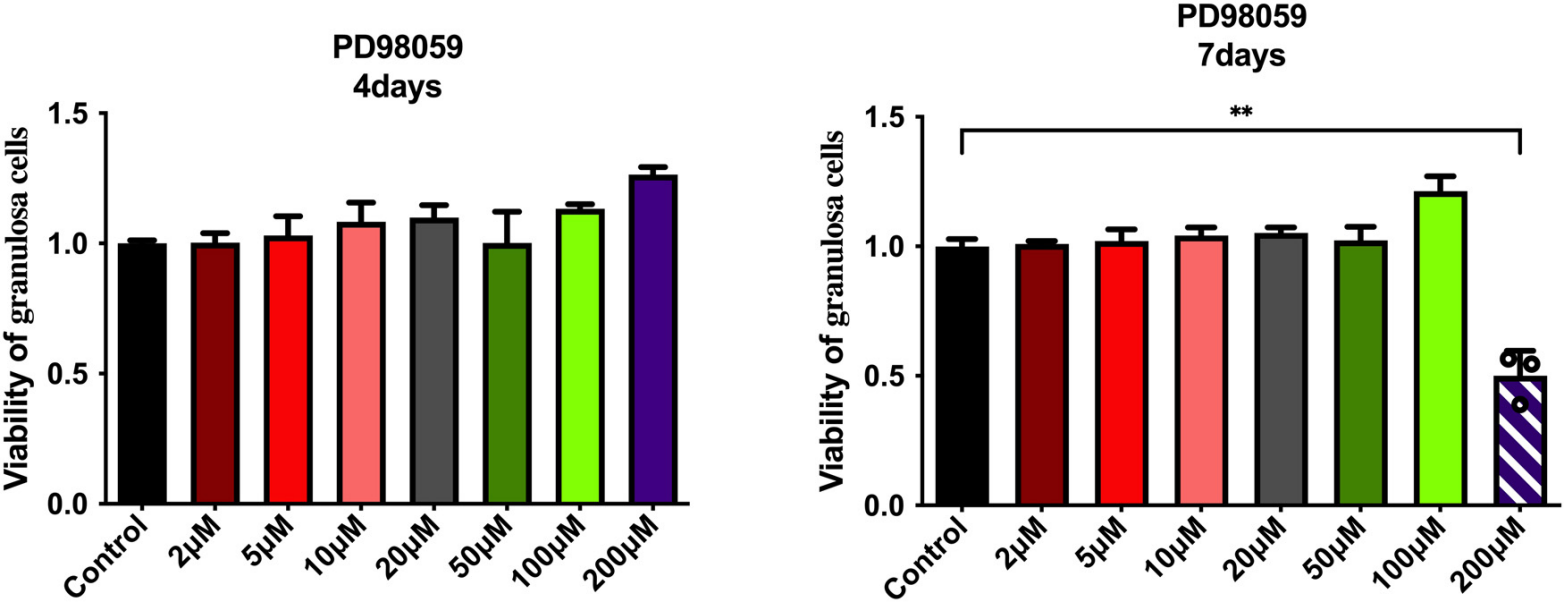
Effect of different concentrations of PD98059 on the viability of granulosa. **P < 0.01 vs control group.

Mitogen-activated protein kinases (MAPK) can be divided into four subgroups: ERK, p38, C-Jun N-terminal kinase (JNK), and ERK5, and ERK becomes active only after phosphorylation. PD98059 is a selective, noncompetitive mitogen-activated protein (MEK) pathway inhibitor that specifically inhibits MEK-1 mediated MAPK activation and dose-dependent inhibition of ERK1/2 phosphorylation. The experimental results did not show any difference in the cell survival rate of ovarian cancer cells treated with different PD98059 concentrations (e.g., 2, 5, 10, 20, 50, 100, and 200 µM) cultured for 4 days or even 7 days. Only the highest concentration of 200 µM PD98059 greatly inhibited granulosa cells, with an inhibition rate of 50%. To sum up, PD98059 had no significant change in the survival rate of ovarian cancer cells within the concentration range of 2–100 µM. According to previous literature, 10 µM of PD98059 could significantly inhibit the ERK signal pathway [21]. Therefore, we selected 10 µM of PD98059 effect on ERK for the next experiments (Figure 7).

### 3.7 HUC-MSCs induced the ERK–Nrf2–HO-1 pathway in PM-stimulated granulosa cells

**Figure 8:**
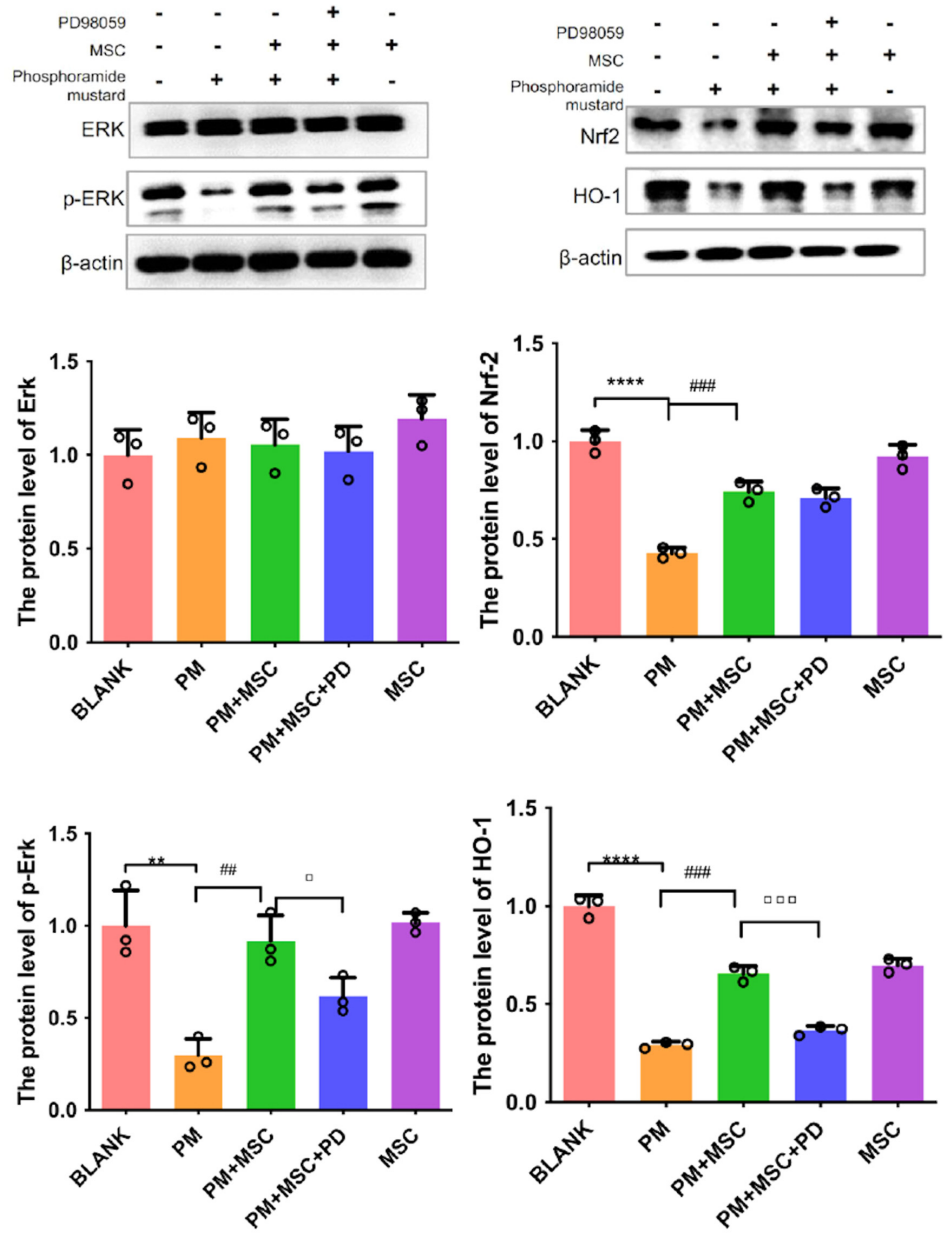
Levels of ERK, p-ERK, HO-1, Nrf2 proteins in granulosa cells in different groups, ****P < 0.0001, **P < 0.001 vs blank group, ^###^ *P* < 0.001, ^##^ *P* < 0.01 vs PM group, ▫▫▫*P* < 0.001, ▫*P* < 0.05 vs PM + MSC group.

**Figure 9:**
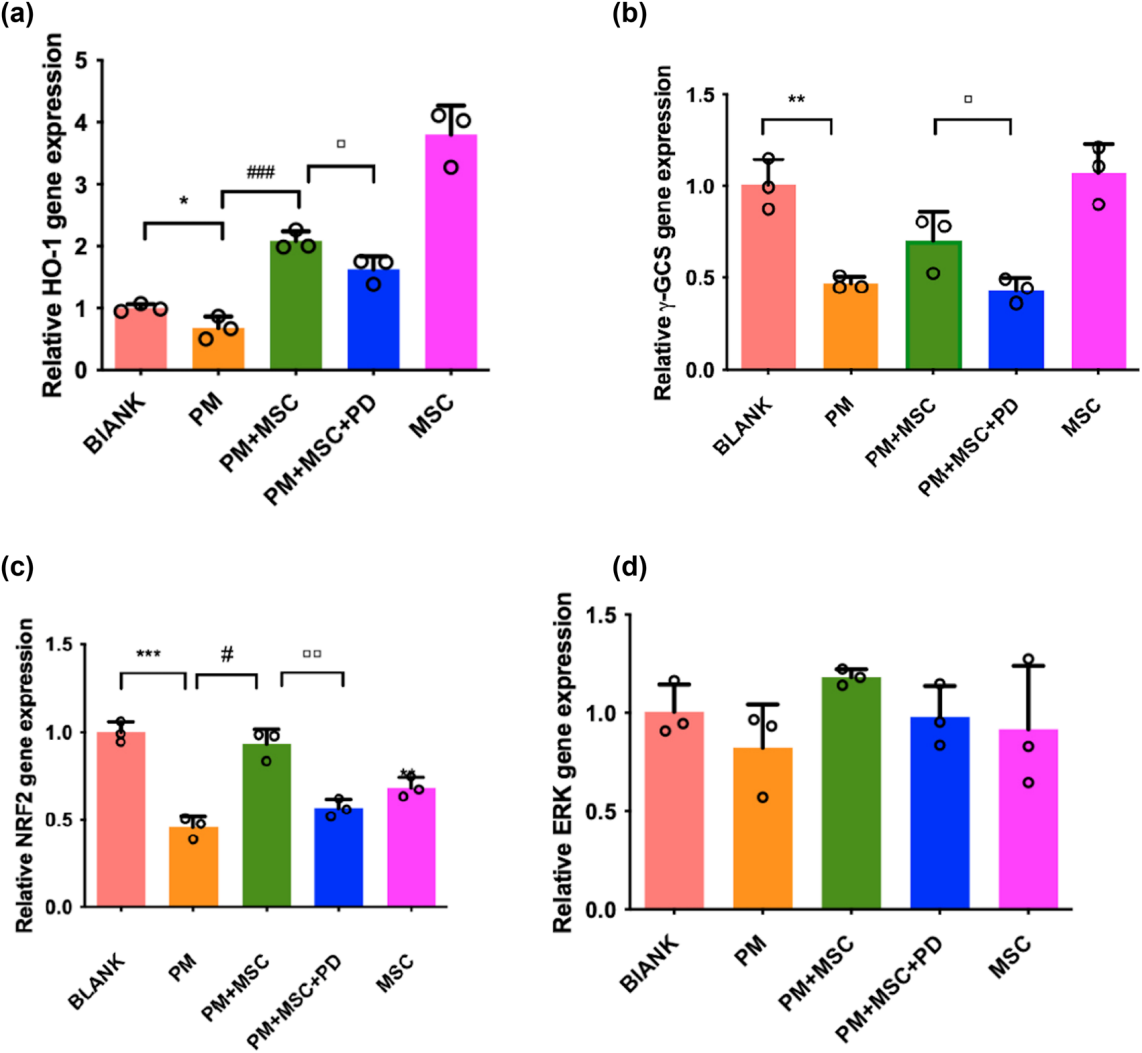
The expression of mRNA in different groups using qPCR analysis. (a) The expression of HO-1 mRNA. (b) The expression of mRNA of γ-GCS mRNA. (c) The expression of Nrf-2 mRNA. (d) The expression of ERK mRNA. ***P < 0.001, **P < 0.01, *P < 0.05 vs blank group, ^###^ *P* < 0.001, ^#^ *P* < 0.05 vs PM group, ▫▫*P* < 0. 01, ▫*P* < 0. 05 vs PM + MSC group.

It was found the protein levels of p-ERK, Nrf2, and HO-1 in ovarian cancer cells tend to decrease after being exposed to PM (*P* < 0.05). After co-culturing with HUC-MSCs, the levels of p-ERK, Nrf2, and HO-1 were increased compared with the model group (Figure 8). In real-time fluorescence quantitative PCR analysis, the expression of HO-1 and Nrf-2 genes decreased after exposure to PM (*P* < 0.05). After co-culturing with HUC-MSCs, the expression of HO-1 and Nrf-2 genes tended to increase (*P* < 0.05) (Figure 9).

### 3.8 Inhibition of ERK damaged the repair effects of HUC-MSCs

After being treated with PD98059, the apoptosis rate of ovarian cancer cells, as well as ROS level (Figure 5a), was increased to a certain extent (*P* < 0.01) (flow cytometry) (Figure 4b), whereas GSH and γ-GCS levels were decreased (Figure 6a and b), as compared with the treatment group. In the qPCR analysis, after being treated with PD98059 the mRNA expression of γ-GCS, HO-1, and Nrf-2 in the PM + MSCs + PD group were decreased than that in the PM + MSCs group (*P* < 0.05) (Figure 9). After being treated with PD98059, the protein levels of p-ERK and HO-1 in PM + MSC groups tend to decrease (*P* < 0.05) (Figure 8). This result indicated that the ERK–Nrf2–HO-1 pathway mediated the protective effects of HUC-MSCs on PM-injured cells.

## 4 Discussion

At present, there is no effective treatment method for POI induced by chemotherapy. MSCs do not cause immune rejection in allogeneic or even xenogeneic transplantation and are widely used in Regenerative Medicine. Previous manuscripts report that MSC can repair tissue damage such as POF [22] and osteoarthritis [23]. In this study, we used CTX active substance PM and ovarian cancer cells to establish an *in vitro* model of granulosa cell chemotherapy injury. Findings show that HUC-MSCs could reduce the OS levels of ovarian cancer cells and reduce the apoptosis of cells, which may be related to the ERK signaling pathway that regulates GSH metabolism, thereby providing a new theoretical basis for HUC-MSCs to treat POI.

In this study, we found that CTX must be metabolized to PM to cause cellular damage. There have been successful cases [24,25] in which CTX was used to build a cell model of chemotherapy damage. We designed to use CTX to make a granulocyte chemotherapy damage model in the experimental design phase. However, the CCK8 assay showed that different CTX concentrations could not cause cell damage. According to the literature review, CTX needs to be oxidized and metabolized into active metabolites *in vivo* to play a role, such as PM and *trans*-4-phenylcyclophosphamide (T4P) [26,27]. We found when the concentration of PM was 50 µM, it would remarkably hinder the proliferation of granulosa cells in a concentration-dependent manner. Therefore, we changed to PM for experimental research in later experiments.

In the study, we found that HUC-MSCs could inhibit PM-induced apoptosis of granulosa cells. Previous studies have shown that MSCs from different sources can repair granulosa cell damage [28,29]. Our research result is consistent with the findings of previous studies.

The repair function of MSCs may be related to OS changes in ovarian cancers. Oxidative stress is an important factor that interrupts normal cell proliferation and apoptosis [30]. It had been confirmed that, in women with polycystic ovary syndrome, the OS of granulosa cells was increased [31]. In our study, we found after HUC-MSCs treatment, the ROS level in injured granulosa cells was decreased compared with the model group (*P* < 0.05), and the level of MDA tended to decrease. Placental MSCs and curcumin can reduce the level of intracellular OS and improve the activity of granulosa cells [32,33]. Umbilical cord MSCs can improve the level of OS in rat granulosa cells [26]. Our research result is consistent with the findings of previous studies.

In this study, the ovary cancer cells and HUC-MSCs were co-cultivated in the study. The repair effect of MSCs may be related to the exosome form of MSCs. Exosomes are related therapies of many diseases, such as polycystic ovary syndrome [34] and POI [11]. Injecting soluble factors from a stem cell culture medium can achieve the same therapeutic effect as stem cell transplantation [35]. In addition, after injecting soluble factors in stem cell culture medium into the target ovary, follicle development was also observed in non-injected ovaries [36]. The exosomes secreted by stem cells may be carriers of paracrine action. Extracellular vesicles are extracellular microtubules that can achieve intercellular dialog through endocytosis and transport proteins, nucleic acids, and other active substances. Research has shown that extracellular vesicles can improve female reproductive function by reducing granulosa cell apoptosis, repairing the endometrium, and reducing endometrial fibrosis and inflammatory response [37].

GSH plays critical roles in protecting cells from oxidative damage, the toxicity of xenobiotic electrophiles, and maintaining redox homeostasis [14,38]. GSH exists as the forms of thiol-reduced and disulfide-oxidized. Most of GSH is in the cytosol, and 10–15% of GSH is in the mitochondria, a very few in the endoplasmic reticulum [39]. The decrease in GSH can modulate cell death in many ways such as apoptosis and fragmentation DNA cleavage [40]. There are a lot of research about GSH metabolism in liver disease, while there is few research about GSH metabolism in granulosa cells, especially in granulosa cell injury. GSH decreasing is a common accompaniment to the damage of granulosa cells [41,42]. γ-GCS is an important rate-limiting enzyme in GSH synthesis [43]. In our study, we found that HUC-MSCs increased GSH levels and decreased GSSG levels in granulosa cells through the expression increase of γ-GCS (Figure 6a).

There were few reports about the signal pathway of GSH in granulosa cells before. One research reported the synthesis of GSH required PI3K, MAPK, and MEK in cardiac myocytes [44]. Agidigbi et al. reported that ERK could regulate GSH syntheses in osteoclasts [45]. ERK1/2 inhibitor AZD0364 could inhibit GSH/GSSG ratio in leukemia cells [46]. Extracellular regulated protein kinases (ERKs), including ERK1 and ERK2, are crucial for transmitting signals from surface receptors to the nucleus. Phosphorylated ERK1/2 translocated from the cytoplasm to the nucleus, participating in various biological reactions such as cell proliferation and differentiation. In this study, we found that HUC-MSCs may regulate the ERK pathway to impact GSH expression. PD98059 is a non-ATP competitive MEK inhibitor. It can specifically inhibit MEK-1-mediated MAPK activation and indirectly inhibit ERK1 and ERK2. Using PD98059, we found the impaction of MSCs on GSH was destroyed. It was consistent with the reports above. Besides, we found that the repair of HUC-MSCs was related to ERK phosphorylation but not ERK (Figure 8). GCLC and GCLM are the key metabolizing enzymes of GSH production and the downstream targets of Nrf2-HO-1 signaling pathway [15]. The transcription factor Nrf2 binding to antioxidant response element 4 (ARE4) was found to be related to human γ-GCS promoter activation [47,48]. The activation of ERK1/2–Nrf2 can up-regulate the expression of HO-1 and reduce the level of cellular OS [49]. In this study, we found after co-culturing with HUC-MSCs, the levels of Nrf2 and HO-1 in ovarian cancer cells increased significantly compared with the model group and decreased after using PD98059. So, we made the conclusion that HUC-MSCs may influence the ERK–Nrf2–HO-1 pathway to increase γ-GCS expression and GSH production. As GSH has a positive effect on the POF cell model, we could use GSH alone in POF therapy or increase the expression of GSH in MSCs to enhance the efficacy of MSC in treating POF. This study found that the repair effects of HUC-MSCs may be related to the ERK–Nrf2–HO-1 pathway to increase GSH production and enhance the antioxidant capacity of granulosa cells, thereby improving the function of granulosa cells and providing a theoretical basis for HUC-MSCs to treat POI. However, this study has some limitations. Many intracellular antioxidant mechanisms still exist. This article focuses on the effects of HUC-MSCs on GSH metabolism, which can subsequently increase the mechanism of HUC-MSCs on superoxide dismutase or NADPH metabolism and further explore the mechanism of HUC-MSCs on the apoptosis of granulosa cells after injury.

In conclusion, this study used PM to act on human granulosa cells to analyze the effects of HUC-MSCs on injured granulosa cells. The results show that HUC-MSCs may regulate the ERK–Nrf2–HO-1 pathway to increase γ-GCS expression and GSH production, reduce ROS level and apoptosis of granulosa cells, and improve antioxidant capacity.
